# Functionalized Antimicrobial Composite Thin Films Printing for Stainless Steel Implant Coatings

**DOI:** 10.3390/molecules21060740

**Published:** 2016-06-09

**Authors:** Laura Floroian, Carmen Ristoscu, Natalia Mihailescu, Irina Negut, Mihaela Badea, Doru Ursutiu, Mariana Carmen Chifiriuc, Iuliana Urzica, Hussien Mohammed Dyia, Coralia Bleotu, Ion N. Mihailescu

**Affiliations:** 1 Faculty of Electrical Engineering and Computer Science, 1 Politehnicii Str., Transilvania University of Brasov, Brasov 500024, Romania; lauraf@unitbv.ro (L.F.); udoru@unitbv.ro (D.U.); 2 National Institute for Laser, Plasma and Radiation Physics, P.O. Box MG-36, Magurele, Ilfov RO-77125, Romania; carmen.ristoscu@inflpr.ro (C.R.); natalia.serban@inflpr.ro (N.M.); negut.irina@inflpr.ro (I.N.); iuliana.iordache@inflpr.ro (I.U.); 3 Faculty of Physics, University of Bucharest, Magurele, Ilfov 077125, Romania; 4 Faculty of Medicine, 56 N. Balcescu Str., Transilvania University of Brasov, Brasov 500019, Romania; mihaela.badea@unitbv.ro; 5 Faculty of Biology, Research Institute of the University of Bucharest–ICUB, University of Bucharest, Spl. Independentei 91-95, Bucharest 050095, Romania; carmen_balotescu@yahoo.com (M.C.C.); mdyia1981@gmail.com (H.M.D.); 6 “Stefan S. Nicolau” Institute of Virology, 285 Mihai Bravu Avenue, Bucharest 30304, Romania; cbleotu@yahoo.com

**Keywords:** functional coatings, MAPLE thin films, antibiotic release, antimicrobial effect

## Abstract

In this work we try to address the large interest existing nowadays in the better understanding of the interaction between microbial biofilms and metallic implants. Our aimed was to identify a new preventive strategy to control drug release, biofilm formation and contamination of medical devices with microbes. The transfer and printing of novel bioactive glass-polymer-antibiotic composites by Matrix-Assisted Pulsed Laser Evaporation into uniform thin films onto 316 L stainless steel substrates of the type used in implants are reported. The targets were prepared by freezing in liquid nitrogen mixtures containing polymer and antibiotic reinforced with bioglass powder. The cryogenic targets were submitted to multipulse evaporation by irradiation with an UV KrF* (λ = 248 nm, τ_FWHM_ ≤ 25 ns) excimer laser source. The prepared structures were analyzed by infrared spectroscopy, scanning electron microscopy, energy dispersive X-ray spectroscopy and profilometry, before and after immersion in physiological fluids. The bioactivity and the release of the antibiotic have been evaluated. We showed that the incorporated antibiotic underwent a gradually dissolution in physiological fluids thus supporting a high local treatment efficiency. Electrochemical measurements including linear sweep voltammetry and impedance spectroscopy studies were carried out to investigate the corrosion resistance of the coatings in physiological environments. The *in vitro* biocompatibility assay using the MG63 mammalian cell line revealed that the obtained nanostructured composite films are non-cytotoxic. The antimicrobial effect of the coatings was tested against *Staphylococcus aureus* and *Escherichia coli* strains, usually present in implant-associated infections. An anti-biofilm activity was evidenced, stronger against *E. coli* than the *S. aureus* strain. The results proved that the applied method allows for the fabrication of implantable biomaterials which shield metal ion release and possess increased biocompatibility and resistance to microbial colonization and biofilm growth.

## 1. Introduction

In recent years, the progress in surgical techniques has led to an explosive growth in the use of biomaterials for advanced medical devices and implants. The implantable biomaterials market was worth $79.1 billion in 2014 and is estimated to grow at 6.73%/year to reach $133 billion in 2022 [1]. It is expected that by 2017 more than five million people will have at least one implanted device. One of the major hazards associated with the introduction of an implant into the human body is the risk of microbial infections. It was found that the advent of bacterial biofilms is of considerable importance in the pathogenesis of medical implant-associated infections [2], which exhibit augmented resistance to both the immune system of the host and antibiotics [3,4,5].

The microbial biofilm is defined as a sessile microbial community adhered to a substrate and protected by an extracellular, self-secreted polymeric matrix, exhibiting an altered phenotype in respect to growth, gene expression and protein production [6]. In this context, *Staphylococcus aureus* is the most common pathogen causing implant-associated infections [7,8]. Moreover, the Gram-negative bacilli also contribute with an important percentage, up to 23%, to this type of infections. In particular, *Escherichia coli* is one of the predominant microorganisms in this respect [9,10,11]. The infections presume the formation of biofilms rendering systemic antibiotherapy and host immune response ineffective and leading to chronic infections, dissemination of biofilm cells to uninfected tissues and eventually implant failure [12]. Antimicrobial coatings are intended to kill a wide range of microbes which can reside on non-living surfaces, as e.g., implants. These antimicrobials are antibacterials, antifungals, antivirals, antiparasitics, or non-pharmaceutical (e.g., essential oils).

One of the most successful approaches in fighting biofilm-associated infections is to prevent the adhesion or to delay the growth of already adhered and/or colonizing microorganisms. To this purpose, the implant surface is coated with metamaterial bioactive films releasing antimicrobial drugs or substances obtained by chemical modification of the biomaterials surface or by tailoring the surface nanostructure of metal implants [13,14,15,16]. Local drug delivery represents an effective and promising procedure to prevent the bacterial adhesion to the implant surface and the formation of microbial biofilms [17,18]. Due to the local release of the drug, a high concentration can be achieved that ultimately reduces the total duration of the antibiotic treatment. Drugs can be loaded on the metamaterial surface by immobilization with chemical bonds [19,20] or by application of passive coatings [21,22] which inhibit bacterial adhesion.

In this context, the fields of medical devices, implants and biomaterials are currently combined for achieving hybrid structures containing biodegradable components, therapeutic drugs and biomolecules that could serve as multifunctional coatings for advanced implants [23,24]. The direct impregnation with antibiotics and immobilization of the antimicrobial agent in a matrix able to bind to a variety of surfaces, as well as the fabrication of antimicrobial active metallic coatings or embedded nanoparticles (Cu, Ag) were thus reported [25,26,27,28,29].

The efficiency of poly(styrene-co-methyl methacrylate) films doped with rifampin, doxycycline and clarithromycin was proved against the biofilm formation by methicillin susceptible and methicillin resistant *S. aureus* strains for up to 21 days [30]. Doxycycline (α-6-deoxy-5-oxytetracyclin) is a broad spectrum, bacteriostatic agent that inhibits protein synthesis by blocking the binding of the aminoacyl-tRNA to the ribosomal acceptor site [31].

The present work explores the potential application of the Matrix-Assisted Pulsed Laser Evaporation (MAPLE) method to print metamaterial bioactive implant coatings containing doxycycline incorporated into a polymer-bioactive glass system to ensure the local drug delivery for preventing and treating implant associated infections. MAPLE is a procedure allowing monolayer thickness control which reproduces the properties and functionality and that can be used for materials which need special protection. Laser radiation acts gently, with no thermal and/or biological degradation and damage, to transfer different compounds, including large molecular weight species, such as polymeric or organic molecules [32].

We thus thought to exploit the antimicrobial properties of doxycycline incorporated into a polymer-bioactive glass system to fight against the widespread of nosocomial or hospital-acquired infections, in a one-step prevention and treatment strategy against implant-associated infections.

The functionalization of a stainless steel implant with nanocomposite thin films consisting of bioactive glasses, ceramics or calcium phosphates was studied in [33,34]. The direct implant application of these materials with good bioactivity is limited because of fragility and reduced mechanical strength at static fatigue, they being thus used in small alveolar reconstruction only. The metamaterial layer is designed to shield the implant against corrosion by body fluids which prove to be surprisingly aggressive. Then, cracks result in the advent of attacked areas and degradation of the implant in contact with physiological fluids. These processes can lead to the release of metallic ions into the body [35] which can be accumulated in inner organs (liver, kidney, spleen) and cause health problems. One should keep in mind that corrosion products of stainless steel (SS) include iron, chromium, nickel and molybdenum, which are toxic and, because of their effects, stainless steel being used only for temporary implants to help bone healing [36,37,38].

The MAPLE transfer of either bioactive glass [39,40] or composite films containing polymers and bioactive glass [41,42,43,44,45] was therefore carried out to avoid the corrosion of implants. In the present work, we tried to extend the transfer of bioactive and anticorrosive coatings of doxycycline (Doxy) added to bioglass (BG) and poly-methyl methacrylate (PMMA), in order to intensively prevent the bacterial adhesion to the implant surface and the formation of microbial biofilms. This way, the risk of implant-associated infection could be limited and eventually eliminated. The antimicrobial activity of the obtained structures against *E. coli* and *S. aureus* was investigated as a case example.

## 2. Results

### 2.1. SEM, Profilometry and Topography Investigations

**Figure 1:**
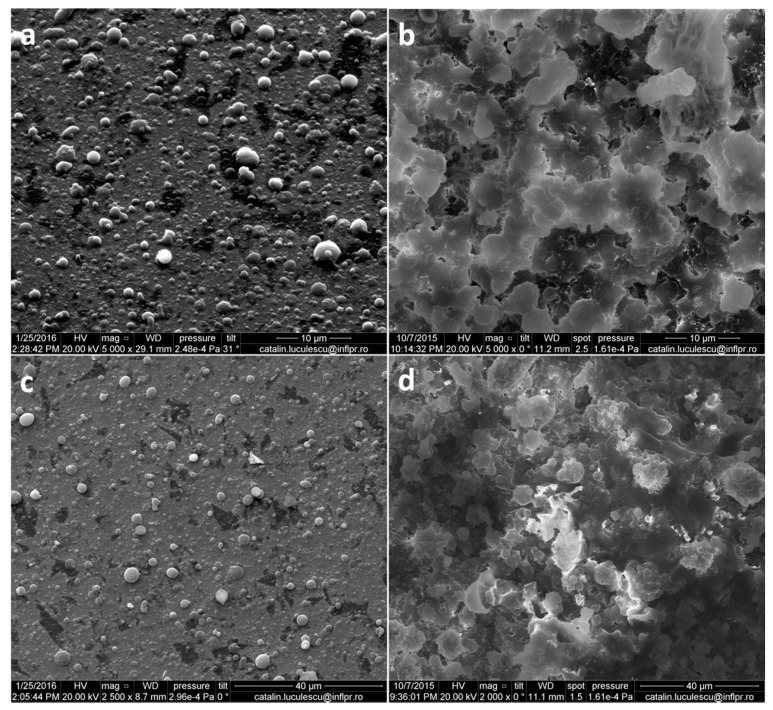
SEM images of BG-PMMA-Doxy before (**a**,**c**) and after 80 days immersion in SBF (**b**,**d**); Images (**c**,**d**) are details of (**a**,**b**), respectively.

The surface morphology of the BG-PMMA-Doxy coatings before and after 80 days immersion in simulated body fluid (SBF) is illustrated in Figure 1.

The films consist of a fairly smooth, dense and homogenous matrix on which isolated and merged spheroidal particulates with an irregular distribution can be observed. Particulates present on surface are in the micrometric range and arbitrarily scattered.

**Figure 2:**
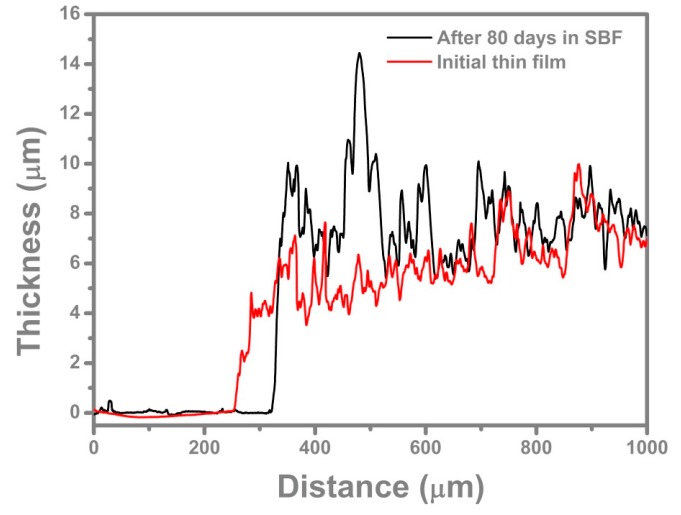
Thickness profile of initial and immersed samples recorded by profilometry.

The profilometry measurements indicated mean values of 530 ± 96 nm for initial thin film thickness and 796 ± 83 nm after 80 days of immersion in SBF (Figure 2). The average values of roughness surface parameters for initial thin films were: Ra = 322 ± 9 nm and Rq = 449 ± 6.5 nm, to become after immersion in SBF: Ra = 329 ± 22 nm and Rq = 433 ± 30.43 nm.

### 2.2. Antibiotic Release Monitored by FTIR and UV-VIS

**Figure 3:**
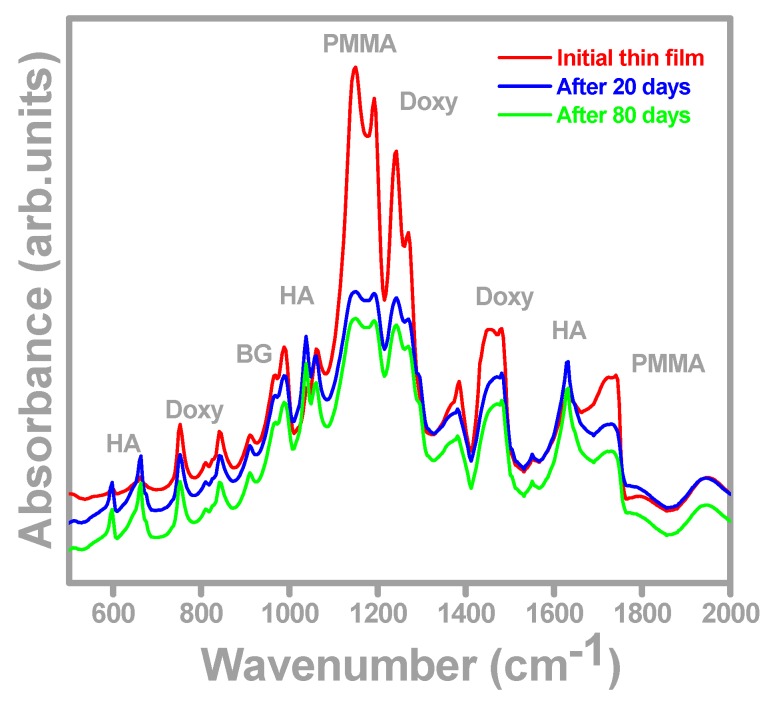
FTIR spectra of initial sample and 20 and 80 days immersion in SBF. The estimated standard deviation is 5.4%.

The FTIR spectra of the freshly prepared films and after 20 and 80 days of immersion in SBF are shown in Figure 3. The spectrum of the initial BG-PMMA-Doxy/SS film (red curve in Figure 3) displays peaks at 1008 and 982 cm^−1^ which correspond to BG. The peaks at 1732, 1244 and 1151 cm^−1^ are assigned to PMMA [46], while the ones at 1456, 1275 and 765 cm^−1^ are representative of Doxy [47]. After 20 and 80 days of immersion in SBF, one can notice significant changes in the FTIR spectrum: a lower amplitude of all peaks compared to the initial one (blue and green curves in Figure 3) together with the occurrence of new peaks at 1648, 1045 and 675 cm^−1^. These peaks belong to carbonated hydroxyapatite (CHA) which is similar to the main component of the bone [48,49].

Thus, FTIR analyses prove, in good accordance with [43,45], that the film reacts with SBF and both chemical and structural changes occur, pointing to the BG decomposition, which most probably promotes the release of Doxy into the surrounding fluid. *In vivo*, one expects this process to allow drug delivery right at the implantation site to prevent implant-associated infections, by reducing the microbe density and their adherence to the implant surface.

In the same time, a new structure self-assembles on the substrate surface, a carbonated hydroxyapatite (CHA) layer. In support of this assertion, the EDS measurements recorded on BG-PMMA-Doxy after 80 days immersion in SBF show a Ca/P atomic ratio of about 1.60, that is in good accordance with [50] (pp. 421–460), which reports values of this ratio within the range 1.38–1.93 for biological apatite. It is to be mentioned [51] that BG-PMMA completely converted to biological apatite after 42 days immersion in SBF.

**Figure 4:**
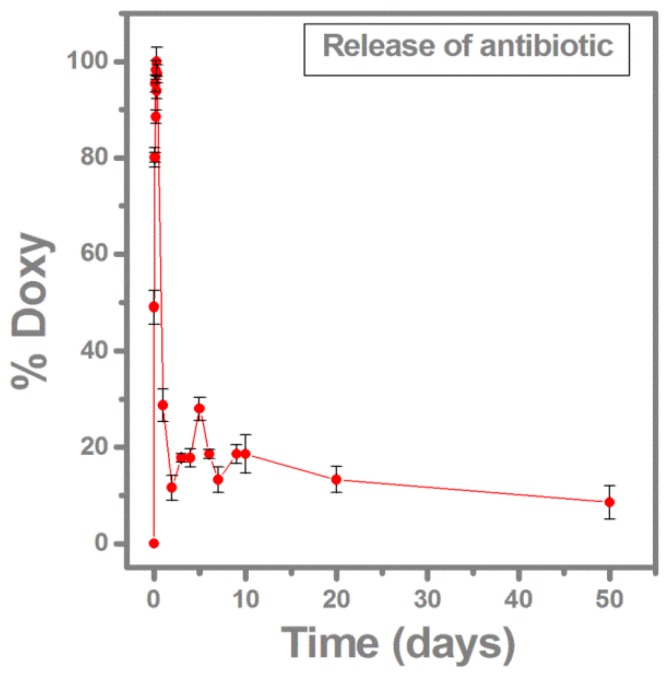
Doxy release into SBF.

The SBF which hosted the samples was investigated in a complementary experiment by UV-Vis spectrometry (Figure 4). Doxy release from the composite into the SBF was detected by the absorbance measurement at 1172 nm wavelength, where the Doxy absorbance shows a maximum.

Figure 4 presents the dynamics of Doxy release into SBF. A rather fast release of the antibiotic can be observed during the first 8 h after soaking, when the maximum Doxy release is reached. Then, a slower deliver stage occurs, releasing 12% of the maximum drug delivered at 2 days of immersion. From this point on, the process becomes almost stationary, at around 20% level. After 50 days, Doxy is still being released from films, confirming our hypothesis that BG-PMMA-Doxy nanostructure assures a prolonged release of the drug.

This evolution is indicative for the gradual BG dissolution in SBF that controls the release of antibiotic molecules from the matrix. It supports a high efficiency of the local treatment *vs.* the oral one as a result of prolonged drug storage in deposited structure.

### 2.3. Electrochemical Polarization Measurements

A batch of six samples: three of bare SS and three of SS covered with BG-PMMA-Doxy films were immersed into 25 mL SBF in sterile polyethylene containers at room temperature and investigated after 0, 7, 14, 21 and 28 days. Initial linear sweep voltammetry (LSV) analysis shows a better corrosion resistance of the nanocomposite layer on SS as compared with bare SS, as characterized by smaller corrosion current and higher corrosion potential (see Table 1).

After 14 and 28 days of immersion in SBF, the measured parameters undergo significant changes, *i.e.*, corrosion current increases and corrosion potential drops. This is indicative of SS corrosion in human fluids. During the same time gap, SS samples covered with BG-PMMA-Doxy thin films ensure a protective behavior against corrosion. The corrosion parameters remain almost unchanged from the initial values and are very different in comparison from the SS samples (Table 1).

Current densities have been normalized to the surface area. The low values of standard deviation signify that the results are reproducible.

**Figure 5:**
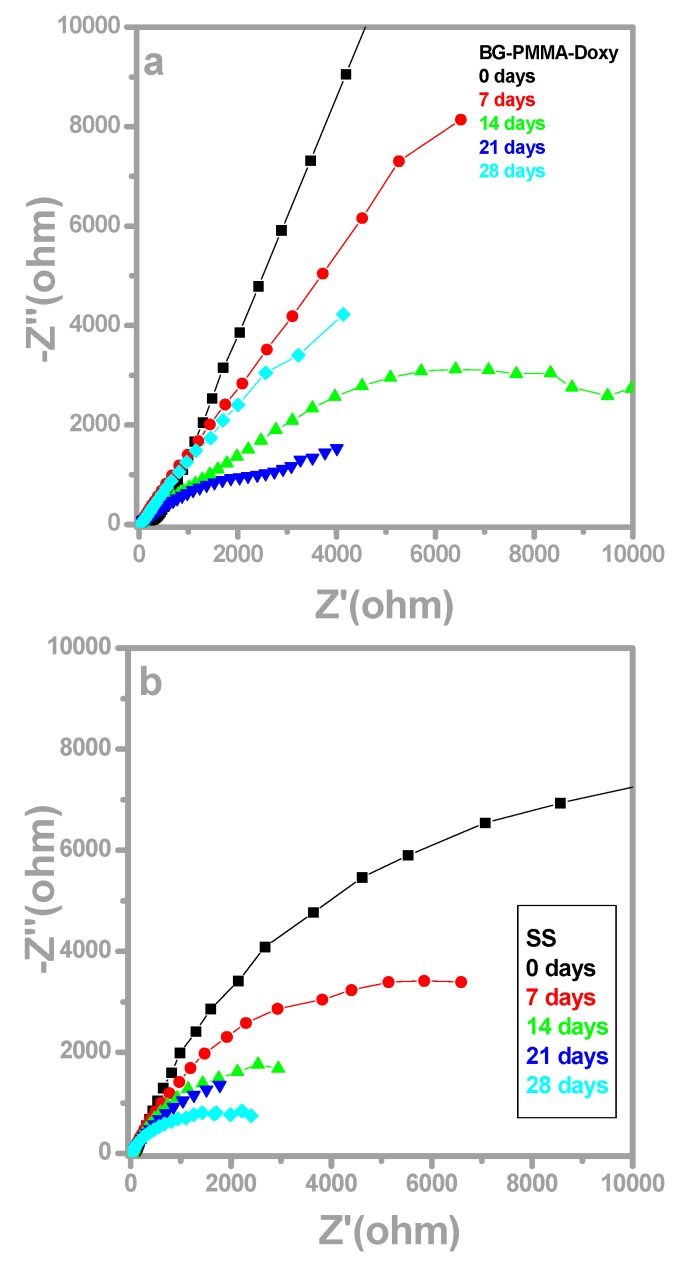
Nyquist diagrams for BG-PMMA-Doxy/SS sample (**a**) and bare SS sample (**b**) after 0 (black), 7 (red), 14 (green), 21 (blue) and 28 (cyan) days of immersion in SBF. The estimated standard deviation is 8.1%.

The electrochemical impedance spectroscopy (EIS) plots (Nyquist diagrams) for the electrodes were recorded in the open circuit potential (OCP) configuration. They exhibited the features of the covering layer and processes at the liquid-solid interface (Figure 5).

**Figure 6:**
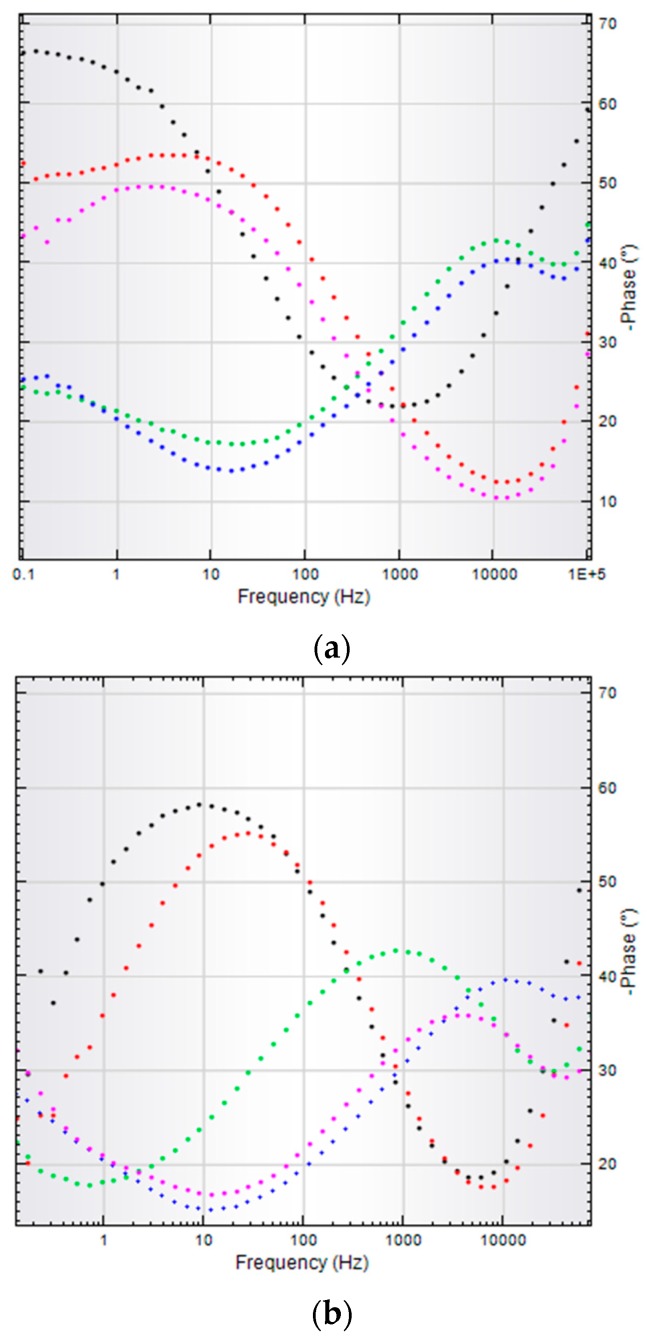
Bode diagrams for BG-PMMA-Doxy/SS thin film (**a**), and for bare SS (**b**) after 0 (black), 7 (red), 14 (green), 21 (blue) and 28 (cyan) days, respectively, of immersion in SBF.

In case of BG-PMMA-Doxy/SS, the substrate is initially covered with a stable, near pure capacitive layer with a high polarization resistance and the corresponding Nyquist diagram (Figure 5a, black curve) is a semicircle with a very large radius, with −67° max phase angle. After 7 days of immersion in SBF, the radius of Nyquist plot slightly decreases (Figure 5a, red curve), while after 14 and 21 days, the Bode plots are characterized by two time constants. Correspondingly, each of them has two max phase angles: −43° and −24° after 14 days and −40° and −28°, after 21 days of immersion, respectively (Figure 6a and Table 2).

A max phase angle close to −45° is characteristic to diffusion process and is indicative for ion exchange between BG-PMMA-Doxy layer and electrolyte, whereas a max phase angle close to −25° points to a simultaneous adsorption process.

### 2.4. In Vitro Biocompatibility Assay

**Figure 7:**
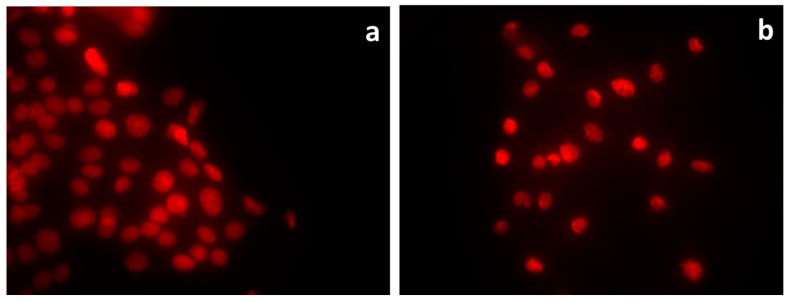
Fluorescence microscopy images of MG63 cells grown for 24 h on BG-PMMA-Doxy/SS thin film (**a**); and (**b**) on control, stained with PI (magnification ×200).

We observed that the morphology and growth of MG63 cells on the obtained thin films were not affected with respect with control (Figure 7). The microscopic results were interpreted in accordance with the recommendations of *ISO 10993-5:2009(E); Part 5. Biological evaluation of medical devices. In vitro cytotoxicity tests*. The examination revealed no changes in the cellular morphology that could be indicative of dead cells, such as round, contracted cells.

**Figure 8:**
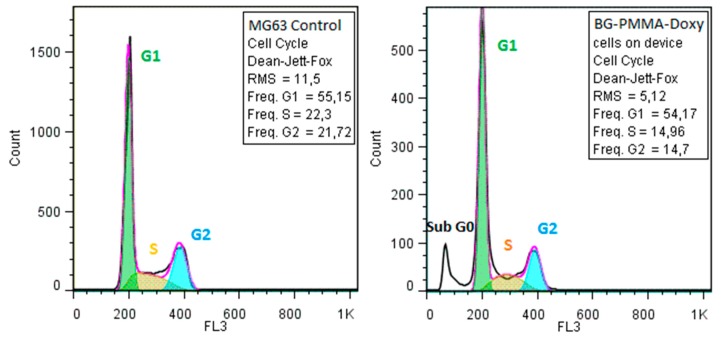
Flow cytometry diagrams of the cellular cycle of MG63 cells grown on BG-PMMA-Doxy thin films.

The microscopic analysis was confirmed by the flow cytometry assay of the cellular cycle, showing no changes in the distribution of the growing phases (Figure 8).

### 2.5. Anti-Biofilm Activity of the Synthesized Thin Films

In order to evaluate both the initial rapid release of the antibiotic in active form, as well as the duration of the antibiotic’s protective action, we assessed the antimicrobial activity within a temporal range varying from 5 min to 24 h. The tested samples inhibited the microbial growth after 5 min of contact with the respective surface. The decrease of the viable cell counts (VCCs) is of more than 1 for *E. coli* and less than 1 log for *S. aureus*. This demonstrates the rapid installation of the bacteriostatic effect of the antibiotic, which stops the multiplication of the viable bacteria present in the initial inoculum.

**Figure 9:**
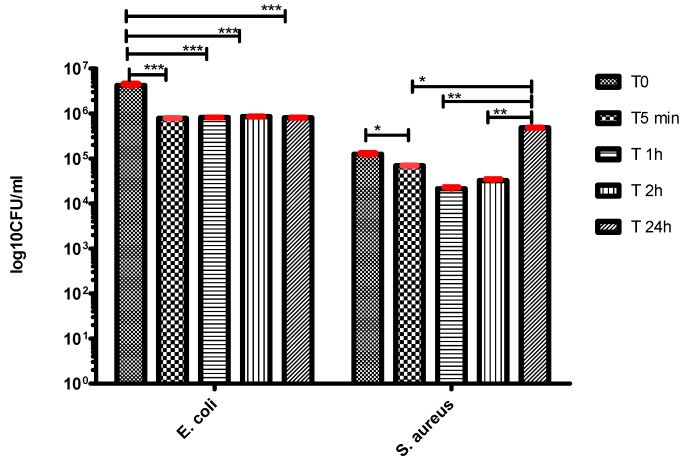
Dynamics of the microbial growth on the BG-PMMA-Doxy thin films (two-way Anova, Bonferroni test, * *p* < 0.05; ** *p* < 0.01; *** *p* < 0.001).

In case of *E. coli*, the microbial growth was inhibited during the entire duration of the experiment, while in case of *S. aureus*, the initial slight decrease of the VCCs observed after 5 min contact, was followed by a more significant one after 1 h of contact, followed by a slight increase after 2 h and an exponential growth till 24 h (Figure 9).

However, in both experimental models, the cellular density keeps inferior to the threshold of 10^6^ CFU/mL, which is considered the minimal infecting dose for the opportunistic bacteria [52]. Below this limit, the host anti-infective defense mechanisms could be effective in eliminating the colonizing bacteria, before the initiation of an infectious process [53].

## 3. Discussion

The spheroidal particulates visible on the top of deposited films are characteristic of the PLD process [50] and proved beneficial for cells adhesion and growth [54,55]. Moreover, the coatings deposited from BG-PMMA-Doxy cryogenic targets on SS substrate have high roughness values (Ra =322 ± 9 nm and Rq = 449 ± 6.5 nm) because they copied the substrate’s morphology. Initial preparation of the SS substrate is very important and should be carefully performed by grinding and sanding with sandpaper grit or immersing in different etching solutions. It has to be mentioned that for orthopedic applications the perfect roughness should be less than 1 mm while for dental ones it should be less than 1 μm. The significant increase of the active area favors biocompatibility by augmentation of cellular adhesion, viability and proliferation [56,57].

The roughness surface parameters, Ra and Rq, were found quite similar for the initial thin film and after 80 days of immersion in SBF. The increased thickness after 80 days of immersion is due, in our opinion, to the synthesis of CHA on the top of the surface along with the releasing of Doxy [51].

Peaks belonging to either PMMA or Doxy were identified in the FTIR spectra. After immersion in SBF, the amplitude of these peaks decreases and new peaks belonging to CHA appear. This conversion is accompanied by controlled Doxy release in SBF which inhibits the microbial biofilms development. The Doxy release is at maximum after 8 h but keeps still significant after 50 days, which confirms the drug reservoir role of the deposited composite coatings.

A major difference between our results in respect with previous reports is related with the dwell time of doxycycline in the polymer—bioglass matrix. Thus, Vester *et al.* [58] report embedding of gentamycin in PDLLA coatings of metallic implants. Nevertheless, the gentamycin is released in a first burst lasting one minute and containing 60% of the incorporated antibiotic, followed by a tail with a much lower intensity. To the contrary, in sharp contrast is the layer’s drug delivery in a progressive manner with a maximum *9 days or more* after implantation, as reported by Schwab *et al.* [59] or Montserrat *et al.* [60].

In our opinion, one should avoid both a rapid or slow release of drug into the body to ensure a high dose of antibiotic in the first 12 h after implantation to prevent the bacterial adhesion and biofilm formation onto the implant surface. We should mention that the half-life of antibiotics is usually 6–18 h.

By comparison, one could value our BG-PMMA-Doxy nanostructure supporting a prolonged release of the drug and a proper concentration on the site of implantation, having an initial progressive and intense discharge with maximum at 8 h and a fairly constant one in the next 10 days.

As known, a material has a better resistance to corrosion whenever it exhibits a lower current and higher potential of corrosion. LSV analyses allow comparing the behavior of the bare SS and covered with BG-PMMA-Doxy nanocomposite samples in contact with human fluids.

Important information is also acquired by EIS analyses. Initially, the Nyquist diagram for bare SS is a semicircle with a smaller radius than in case of BG-PMMA-Doxy/SS sample, which indicates a poor resistance against corrosion of SS. The radius of Nyquist diagram falls with the duration of immersion, pointing to an inappropriate behavior of SS in human fluids. The max phase angle in Bode diagrams also decreases and a single time constant indicates that a unique diffusion process takes place, namely SS degradation by corrosion in the body environment.

The two time constants characteristic to Bode plots of BG-PMMA-Doxy/SS samples mean that two processes take place simultaneously: particle diffusion from the covering layer to the liquid (−43° max phase angle) and adsorption of ions from SBF into the sample (−24° max phase angle), in accordance with SEM and FTIR analyses. Adsorption of ions from SBF induces the conversion of BG in CHA on sample surface, as supported by FTIR and EDS studies. Our FTIR analyses also reveal the release of antibiotic concurrently with glass’ ions.

After 28 days of immersion in SBF, one semicircle Nyquist plot and Bode diagram with a single time constant (−51° max phase angle) were obtained. The amorphous CaO-P_2_O_5_ film crystallizes as an effect of OH^−^ and CO_3_^2−^ ion incorporation and the CHA layer is forming. This protective insulator layer stops ion transfer and possesses a capacitive behavior that remains at the origin of the Nyquist plot modification.

An implant should be fabricated of biocompatible materials that do not cause rejection reactions and allow the implant osseointegration/biointegration with the bone. Often, special coatings are designed in order to increase the adherence of the implant to the bone tissue [61]. In our case, the lack of dead MG63 cells with no modified morphology proved the absence of cytotoxicity of the deposited coatings.

Implant infections represent a major complication in reconstructive surgery. Despite the recent progress of implant surgical techniques to decrease the risk of infections, once implant infection is diagnosed, this can often lead to the surgical removal and need of a strong antimicrobial therapeutic regime. As known, *S. aureus* and *E. coli* are among the most important pathogens in nosocomial infections associated with medical implants [62,63]. The development of efficient anti-microbial implant coatings was recently proposed to mitigate the challenge of biofilm associated infections [64]. In our study, the dynamics of the microbial growth was different for the two tested strains: *S. aureus* and *E. coli*. This difference can be accounted by the variance of generation time and affinities between antibiotic and the molecular target in the two experimental models. It had been suggested that in the presence of certain growth limiting factors, such as osmotic stress, the Gram-negative bacteria adapt more slowly as compared to Gram-positive ones, as revealed by the relative lag time and therefore the time required by bacteria to adapt to novel environmental conditions [65,66].

Accordingly, the quick adaptation of *S. aureus* to the antibiotic containing medium, promotes the more rapid multiplication and consumption of the antibiotic, explaining the increase of *S. aureus* density after 2 h of incubation.

These results demonstrate that the printed thin films are resistant to microbial colonization and can stay at the origin of a significant delay in the microbial biofilm initiation and further development.

We expect the implants coated with BG-PMMA-Doxy metamaterials by MAPLE be soon customized irrespective of the required geometry. The new implant generation will simultaneously meet, due to the special coating, the requirements of hampering the release of metal ions and the formation of microbial biofilms which could irreversibly infect the fabricated bio-product. We mention in this respect that the pulsed laser deposition procedure was already used to coat a customized screw implant [67]. In another paper, Jardini *et al.* [68] used the radiographic CT-scan and mechanical engineering design and manufacturing of real sample to cover dental screws or 3D printed customized implants with different layers.

## 4. Materials and Methods

### 4.1. Materials and Experiment

Medical grade stainless steel type 316L plates (denoted SS) were used as substrates for the film deposition. They contain 64.26 wt % Fe, 18.51 wt % Cr, 12 wt % Ni, 2.13 wt % Mo, 1.44 wt % Mn, 0.58 wt % Cu, 0.56 wt % Si, 0.0265 wt % C, 0.0036 wt % S and other elements in smaller concentration. Simple and coated SS with nanostructures containing bioactive glass (BG), polymer and antibiotic were investigated for physical and chemical parameters and then submitted to biological assays.

Prior to deposition, (1 × 1) cm^2^ SS plates were mechanically processed by polishing to a roughness within μm range (Rq = 2–4 μm), cleaned with acetone, ethanol and deionized water in an X-Tra 30 H ultrasonic bath. An appropriate surface morphology provides a good cell adhesion and bone growth while a suitable roughness insures a long term, enhanced interaction to bone-implant [54,55].

BG is a SiO_2_-Na_2_O-K_2_O-CaO-MgO-P_2_O_5_ system and exhibits very good bioactivity and biocompatibility properties. It contains 56.5% SiO_2_, 11% Na_2_O, 3% K_2_O, 15% CaO, 8.5% MgO, 6% P_2_O_5_ in wt % and was fabricated according to a protocol described in [69,70]. Poly (methyl methacrylate) (PMMA) is an inert polymer that shows good biocompatibility with the human tissue, able to protect the metallic implant against degradation processes [71].

For this study, we selected doxycycline (Doxy), an antibiotic belonging to the tetracycline group [72]. Doxy is known to be stable at body temperature and water soluble, able to ensure diffusion from the carrier. It is active against the most common bacterial pathogens involved in bone and soft tissue infections, and is locally released at concentrations exceeding up to ten times the minimum inhibitory concentration for the concerned pathogens. The Doxy capsules used in our experiments were commercially available and contained 100 mg doxycycline hyclate each.

A matrix consisting in a solution of 0.6 g PMMA dissolved in 19.3 mL chloroform with the addition of 0.08 g BG and 0.11 g doxycycline hyclate was used as a frozen target in MAPLE deposition [73], according to a protocol described in [43]. The MAPLE method is used in case of polymers and delicate substances because it faithfully replicates the properties and functionality of the starting materials, with no photochemical decomposition and damage under the direct action of intense UV laser pulses [41,74,75,76]. All the experiments were conducted using a KrF* (λ = 248 nm, τ_FWHM_ ≤ 25 ns) excimer laser source which was operated at fluence of 0.55 J/cm^2^ and a repetition rate of 5 Hz. For the growth of one thin film, 5000 subsequent laser pulses were applied. In order to obtain a uniform layer and to avoid drilling, the target and substrate were continuously rotated at 50 rpm, while the background pressure inside the deposition chamber was of 2 × 10^−2^ mbar. During the deposition, the target was kept at liquid nitrogen temperature using a cryogenic rotating setting. Depending of their further characterization type, thin films were deposited on Si <100> or SS substrates.

### 4.2. Physical–Chemical Characterization of Deposited Thin Films

#### 4.2.1. Morphological Examination

The morphological characterization was carried out by scanning electron microscopy (SEM) using an XL30 electron microscope (Philips, Edinburgh, The Netherlands) operating at 20 kV acceleration voltage, in high vacuum, under secondary electron mode.

The coating thickness and topography were monitored and estimated by profilometry using a stylus profiler XP2 from Ambios Technology (Santa Cruz, CA, USA); 0.01 mm/s speed; 1 mm working distance for thickness measurements, and 5 mm working distance for roughness measurement and range 10 μm. For this investigation, a short zone of SS substrate was shielded with tape during deposition in order to evaluate the level difference between top of the coating and uncoated SS. For statistics, we have performed measurements on three identical samples.

#### 4.2.2. Composition

Energy Dispersive X-ray Spectroscopy (EDS) studies were carried out on all specimens using a SiLi detector (EDAX Inc., Philips, Edinburgh, The Netherlands) operated at 20 kV. The investigations were performed in triplicate, on relatively large areas of (250 × 250) μm^2^.

The chemical structure of the coatings was investigated by Fourier Transform Infrared Spectroscopy (FTIR). FTIR spectra were acquired with a 8400S instrument (Shimadzu Corp, Kyoto, Japan) which operates in the range of 7800–350 cm^−1^, with spectral resolution of 0.4 cm^−1^ and S/N ratio 20,000:1. The spectra were recorded in absorbance mode.

The Simulated Body Fluid (SBF) containing the release products of Doxy was analyzed using a Cintra10e UV–VIS–NIR spectrophotometer (GBC Scientific Equipment Pty Ltd., Melbourne, Australia), in the (190–1200) nm range.

SBF has an ionic composition identical to blood plasma and was prepared after Kokubo’s formula [77] by mixing the proper reagents (Table 3).

Both bare controls of uncovered SS and covered SS with BG-PMMA-Doxy thin film were immersed into 25 mL of SBF in sterile polyethylene containers.

The samples were kept at a temperature of 37 °C using a Binder Microbiological Incubator and their surface was investigated after different immersion times by FTIR. All measurements were conducted in triplicate, in accordance with the ISO/FDIS 23317:2007(E) Standard.

#### 4.2.3. Electrochemical Investigation

Electrochemical measurements were performed with an Autolab PGSTAT 100 system (Eco Chemie, Eco Chemie, Utrecht, The Netherlands) controlled by Nova software, in a three electrodes configuration with Ag/AgCl reference electrode and platinum wire as counter electrode. Corrosion resistance in SBF of samples used as coatings for implants or prosthesis was assessed using electrochemical methods because of their high sensibility. The influence of physiological fluids on SS bare substrates and BG-PMMA-Doxy/SS was firstly studied by linear sweep voltammetry that can characterize corrosion mechanisms and predict corrosion rates. There is a linear relationship between the metal dissolution or corrosion rates, and the corrosion current, therefore they are important parameters which can be found by LSV. The plots were recorded between −1.50 and −0.25 V with 0.1 V/s scan rate. The measurements for three samples of each studied type have been conducted in order to obtain reliable results. The mean values of corrosion parameters (corrosion potential (E_corr_) and corrosion current (i_corr_)) were inferred.

Moreover, electrochemical parameters of the induced processes were estimated by electrochemical impedance spectroscopy. 0.01 V amplitude sinusoidal voltage perturbations was applied, scanning from 10,000 to 0.1 Hz with 10 points per frequency decade and auto-integration time 5 s, while the samples were immersed in SBF used as electrolyte. The working electrodes are square and have a 0.75 cm^2^ active surface area.

### 4.3. Biological Assays

#### 4.3.1. Biocompatibility

The biocompatibility of the obtained films deposited on SS substrates was evaluated *in vitro* on human bone osteosarcoma (MG63, ATCC^®^ CRL-1427™) cells, by investigating the morphology and cellular cycle of the cells growing on the obtained thin films. For microscopic evaluation of cellular morphology the obtained coated specimens were placed in 24-well plates exposing the coated sample surface. 5 × 10^5^ MG63 cells were added in Dulbecco’s Modified Eagle’s Medium (DMEM) supplemented with 10% fetal bovine serum and 1% essential amino acids. The plates were maintained for 24 h at 37 °C in 5% CO_2_. After 24 h, the samples were stained with propidium iodide (PI), immediately visualized in fluorescence with a FC450C microscope (Leica Microsystems IR GmbH, CH-9435 Heerbrugg, Switzerland) and photographed.

For the cellular cycle assay, the MG63 cells were cultivated in Roswell Park Memorial Institute (RPMI) 1640 medium (Gibco, New York, NY, USA) supplemented with 10% heat-inactivated bovine serum and penicillin/streptomycin and incubated at 37 °C in 5% CO_2_ for 24 h. Then, the monolayers were harvested, washed with phosphate buffered saline (PBS), fixed in 70% cold ethanol and incubated overnight at −20 °C. Each sample was washed in PBS, treated with 100 μg/mL RNase A for 15 min, stained with10 μg/mL PI by incubation at 3 °C for 1 h. Then, the events acquisition was performed with an Epics XL flow cytometer (Beckman Coulter, Inc., 250 S. Kraemer Blvd. Brea, CA 92821, USA). The obtained data were analyzed using the FlowJo software (Version -7.2.5, FlowJo, LLC, Ashland, OR, USA) and expressed as fractions of cells in different cycle phases.

#### 4.3.2. Antimicrobial Biofilm Activity

The anti-biofilm activity of obtained samples was investigated using *S. aureus* ATCC 6538 and *E. coli* ATCC 8739 strains. The tested samples were sterilized by exposure to UV for 30 min. After UV sterilization, an amount of 20 μL of microbial suspension of 0.5 MacFarland density prepared from fresh cultures developed on tryptic soy agar (TSA) was distributed over the samples treated surface and incubated at 37 °C in humid atmosphere for 5 min, 1, 2 and 24 h. After each incubation period, the samples were suspended in 5 mL sterile saline and vortexed vigorously to re-suspend the adherent bacteria. Then, serial ten-fold dilutions were carried out from the recovered suspension, divided on solid medium in triplicate spots of 10 μL each and the VCCs were determined and expressed as colony forming units (CFU)/mL. Statistical analysis was performed using the GraphPad Prism Software, Version 4.00 for Windows, GraphPad Software, San Diego, CA USA, www.graphpad.com.

## 5. Conclusions

Thin films of BG-PMMA-Doxy composite were deposited (imprinted) on 316 L Stainless Steel substrates by the MAPLE technique. When in contact with body fluids, the films demonstrate the ability to stimulate the growth of biological hydroxyapatite on their surface, which validates the film bioactivity. The BG dissolution in human body fluids is accompanied by a prolonged release of active Doxy molecules, an ideal circumstance for the prevention of local infections. Both polymer and apatite layers that grow on the implant surface four weeks after samples immersion into simulated body fluid ensure a good protection against degradation and release of harmful metallic ions (Cr, Ni, Cu) into the body. The printed structures are highly biocompatible and resistant to microbial colonization and can induce a significant delay in the microbial biofilm initiation and their further development.

## Figures and Tables

**Table 1:** Corrosion parameters after different immersion times.

Sample	TIME	i_corr_ (μA/cm^2^)	E_corr_ (mV)
SS	0 days	15.38 ± 0.41	−625.45 ± 0.32
	14 days	18.22 ± 0.12	−773.91 ± 0.21
	28 days	25.14 ± 0.34	−997.38 ± 0.22
BG-PMMA-Doxy/SS	0 days	6.21 ± 0.12	−389.02 ± 0.34
	14 days	7.15 ± 0.31	−425.18 ± 0.10
	28 days	6.96 ± 0.07	−420.03 ± 0.12

**Table 2:** Max phase angle after different immersion times.

Sample	Time (Days)	Max Phase Angle (deg)	
SS	0	−58 ± 4	
	7	−55 ± 2	
	14	−43 ± 2	
	21	−40 ± 4	
	28	−36 ± 2	
BG-PMMA-Doxy/SS	0	−67 ± 4	
	7	−54 ± 4	
	14	−43 ± 3	−24 ± 2
	21	−40 ± 3	−28 ± 2
	28	−50 ± 3	

**Table 3:** Ion concentrations of SBF *vs.* plasma blood [77].

Ions	Na^+^	K^+^	Mg^2+^	Ca^2+^	Cl^−^	HPO_4_^2−^	SO_4_^2−^	HCO_3_^−^
Composition (mM)	142	5	1.5	2.5	147.8	1	0.5	4.2

## Abbreviations

The following abbreviations are used in this manuscript:

MAPLE	Matrix-Assisted Pulsed Laser Evaporation
PLD	Pulsed Laser Deposition
CHA	Carbonated Hydroxyapatite
VCCs	Viable cell counts
SS	Stainless steel type 316L plates
BG	Bioactive glass
PMMA	Poly (methyl methacrylate)
Doxy	Doxycycline
SEM	Scanning Electron Microscopy
EDS	Energy Dispersive X-ray Spectroscopy
FTIR	Fourier Transformed Infrared Spectroscopy
SBF	Simulated Body Fluid
LSW	Linear sweep voltammetry
E_corr_	Corrosion potential
i_corr_	Corrosion current
EIS	Electrochemical impedance spectroscopy
MG63	Human bone osteosarcoma cells
DMEM	Dulbecco’s Modified Eagle’s Medium
PI	Propidium Iodide
RPMI	Roswell Park Memorial Institute
PBS	Phosphate buffered saline
tRNA	Transfer ribonucleic acid
TSA	Tryptic soy agar
